# Massive Hematometra due to Congenital Cervicovaginal Agenesis in an Adolescent Girl Treated by Hysterectomy: A Case Report

**DOI:** 10.1155/2013/640214

**Published:** 2013-02-28

**Authors:** Turki Gasim, Fathia E. Al Jama

**Affiliations:** Department of Obstetrics and Gynecology, College of Medicine, University of Dammam, Dammam, Saudi Arabia

## Abstract

A case of massive hematometra with a bicornuate uterus in a 14-year-old mentally handicapped girl complicated by vaginal agenesis and absent cervix is presented. She was managed by abdominal hysterectomy and right salpingo-oophorectomy that included the ovarian cystadenoma. The left ovary was conserved. This treatment was considered appropriate for this patient.

## 1. Introduction

The independent occurrence of congenital hematometra without hematocolpos is a rare Mullerian anomaly which results from a noncommunicating rudimentary horn with functioning endometrium or primary cervical atresia and absent upper vagina [1–3].

We report a case of massive hematometra, right-sided hematosalpinx and a right ovarian tumor in a 14-year-old premenarcheal, mentally retarded girl who presented with acute abdominal pain and a pelviabdominal mass reaching the xiphisternum. This paper was approved by the hospital research and ethical committee.

## 2. Case Report

This 14-year-old girl was referred to our hospital from a peripheral gynecologic unit. She was mentally dull and noncommunicative. The mother stated that for the past 18 months the patient used to cry every month for a few days with abdominal pain. She was found to hit her abdomen with the pain in the recent months. Initially, the pain was relieved with oral analgesics, but later she required parenteral analgesics for pain relief. Prior to her referral to the hospital, she was reviewed by a urologist in another hospital who found a right-sided hydronephrosis and hydroureter and treated her with a double J-stent which relieved her loin pain.

The mother noticed gradual abdominal enlargement in her daughter who had never menstruated.

On abdominal examination in our unit, a pelviabdominal mass reaching the xiphisternum was found which was tense and tender with a smooth contour.

An ultrasound scan of the pelvis and abdomen revealed a blind upper vagina and bicornuate uterus with the right horn containing the hematometra and fundus reaching the epigastrium. The left horn was rudimentary and noncommunicating with the endometrial cavity. There was a right hemorrhagic tuboovarian mass 7 cm × 7 cm with multiple cysts in the ovary. The left ovary appeared normal.

Both the kidneys and ureters were normal. Pelviabdominal magnetic resonance imaging (MRI) confirmed the above findings. Intravenous pyelography was within normal limits.

A decision to undertake a laparotomy was taken to relieve her pain caused by the hematometra. This was explained to the parents, and written consent was obtained for possible hysterectomy.

Examination under anesthesia revealed a blind lower vagina that measured 1.3 cm. On rectal examination the pelvis was tense and filled with the lower end of the hematometra. As vaginal drainage of the hematometra was impossible, the abdomen was opened by a vertical midline incision reaching up to 3 cm above the umbilicus. Manual exploration of the mass revealed a bicornuate uterus with a rudimentary horn on the left side and a normal ovary. The right cornu was massively distended reaching the xiphisternum. There was gross hematosalpinx on the right side glued together with an enlarged ovary forming a tuboovarian mass measuring approximately 8 cm × 8 cm.

**Figure 1:**
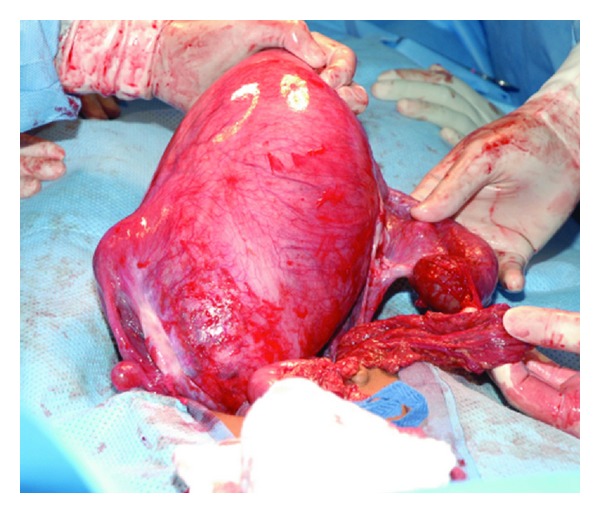
Enlarged right uterine cornu after evacuation of the hematometra.

As it was not possible to deliver the uterus, it was decided to drain the hematometra by a midline bore metallic suction drain which was inserted in the upper anterior uterine wall. At least 2 liters of tarry fluid was drained. This shrunk the conical uterine mass to one-third its original size and could then be delivered through the abdominal incision (Figure 1). No cervix and vaginal could be palpated.

**Figure 2:**
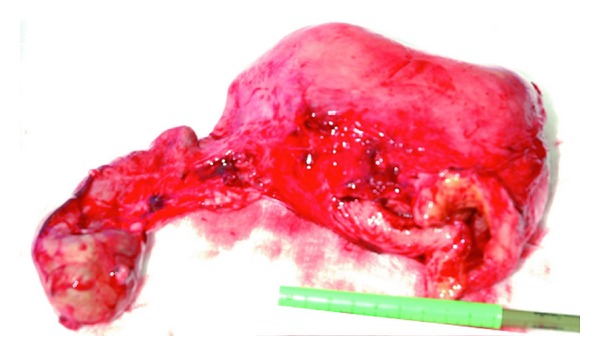
Hysterectomy specimen without the cervix and right ovarian mucinous cyst adenoma (8 cm × 7 cm).

Routine hysterectomy along with the left rudimentary horn and right salpingo-oophorectomy was carried out (Figure 2). The left ovary was conserved. The patient had an uneventful postoperative recovery. She was allowed home on the 7th day after operation. Histopathology reported the right ovarian mass to be mucinous cystadenoma (8 cm × 7 cm) and a rudimentary left horn of the uterus with nonfunctioning endometrium.

## 3. Discussion

Complete cervicovaginal agenesis with a functioning endometrium in bicornuate uterus is an extremely rare Mullerian duct malformation [4]. Only few cases of such abnormality have been reported along with their surgical procedures [5–8].

This mentally retarded, 14-year-old girl had cyclical abdominal pain for the past 18 months expressed by her hitting the abdomen. As she was in the perimenarcheal age and presented with an abdominal mass, it was suspected to be due to cryptomenorrhea resulting from entrapped menstrual blood in the uterine cavity causing pain. A pelviabdominal ultrasound scan showed hematometra. MRI confirmed the absent cervix and upper vagina.

Our case highlights that Mullerian duct anomalies should be considered amongst the differential diagnosis of cyclical abdominal pain that responds poorly to analgesics. As developmental anomalies of the urinary and Mullerian tracts are commonly associated, the former anomaly should be specifically investigated for before elective surgery is carried out.

Surgical interventions for the simpler Mullerian duct malformations such as imperforate hymen, transverse vaginal septum [9, 10], and cervical atresia [2, 11] have been performed without complications. Creation of the new vagina/cervix requires more complex operations [3, 5, 6, 8] associated with high morbidity and limited success, many of these patients ultimately requiring hysterectomy. In this patient reconstructive surgery was thought to be unsuitable because of the associated morbidity. The general consensus of treatment of these patients has been to remove the Mullerian structures during the initial operation so as to avoid postoperative complications. The same principle of treatment was applied in the case described.

## 4. Conclusion

Although technical advances favor reconstructive surgery for cervicovaginal agenesis, it must be remembered that these complex procedures are not without complications resulting in recurrence of hematometra ultimately requiring hysterectomy. In this patient the decision for hysterectomy was considered most appropriate to suit her social circumstances.

## References

[1] Rana A, Gurung G, Begum SH, Adhikari S, Neupane BB (2008). Hysterectomy for hematometra in a 15-year-old mentally handicapped girl with congenital cervicovaginal agenesis and concomitant ovarian adenoma. *Journal of Obstetrics and Gynaecology Research*.

[2] Garat JM, Martinez E, Aragona F, Gosalbez R (1984). Cervical uterine atresia with hematometra: a rare cause of urinary retention in a girl. *Journal of Urology*.

[3] Khunda SS, Al-Omari S (1998). A new approach in the management of lower Mullerian atresia. *Journal of Obstetrics and Gynaecology*.

[4] Goluda M, Gabrys MS, Ujec M, Jedryka M, Goluda C (2006). Bicornuate rudimentary uterine horns with functioning endometrium and complete cervical-vaginal agenesis coexisting with ovarian endometriosis: a case report. *Fertility and Sterility*.

[5] Bugmann P, Amaudruz M, Hanquinet S, La Scala G, Birraux J, Le Coultre C (2002). Uterocervicplasty with bladder mucosa for the treatment of complete cervical agenesis. *Fertility and Sterility*.

[6] Gurbuz A, Karateke A, Haliloglu B (2005). Abdominal surgical approach to a case of complete cervical and partial vaginal agenesis. *Fertility and Sterility*.

[7] Mhaskar R (2005). Amniotic membrane for cervical reconstruction. *International Journal of Gynecology & Obstetrics*.

[8] Lee CL, Wang CJ, Liu YH, Yen CF, Lai YL, Soong YK (1999). Laparoscopically assisted full thickness skin graft for reconstruction in congenital agenesis of vagina and uterine cervix. *Human Reproduction*.

[9] Oga M, Anai T, Yoshimatsu J, Kawano Y, Hayata T, Miyakawa I (1992). Retrohymenal vaginal atresia with perforate transverse vaginal septum. *Gynecologic and Obstetric Investigation*.

[10] Quinn T, Erickson V, Knudson MM (2001). Down's syndrome, precocious puberty, and transverse vaginal septum: an unusual cause of abdominal pain. *Journal of Pediatric Surgery*.

[11] Alborzi S, Momtahan M, Parsanezhad ME, Yazdani M (2005). Successful treatment of cervical aplasia using a peritoneal graft. *International Journal of Gynecology and Obstetrics*.

